# There is a significantly inverse relationship between dietary riboflavin intake and prevalence of osteoporosis in women but not in men: Results from the TCLSIH cohort study

**DOI:** 10.3389/fnut.2023.1112028

**Published:** 2023-02-07

**Authors:** Min Wan, Hongmei Wu, Xuena Wang, Yeqing Gu, Ge Meng, Qing Zhang, Li Liu, Juanjuan Zhang, Shaomei Sun, Qiyu Jia, Kun Song, Weina Gao, Zhanxin Yao, Kaijun Niu, Changjiang Guo

**Affiliations:** ^1^Tianjin Institute of Environmental and Operational Medicine, Tianjin, China; ^2^Nutritional Epidemiology Institute and School of Public Health, Tianjin Medical University, Tianjin, China; ^3^Nutrition and Radiation Epidemiology Research Center, Institute of Radiation Medicine, Chinese Academy of Medical Sciences & Peking Union Medical College, Tianjin, China; ^4^Health Management Centre, Tianjin Medical University General Hospital, Tianjin, China

**Keywords:** dietary factor, riboflavin, osteoporosis, bone mineral density, cross-sectional study

## Abstract

**Background:**

Epidemiological evidence for the relationship between riboflavin intake and bone health is inconsistent, and this relationship has not been examined in Chinese population. This study aimed to investigate the relationship between dietary intake of riboflavin and prevalence of osteoporosis in a Chinese adult population.

**Methods:**

A total of 5,607 participants (mean age, 61.2 years; males, 34.4%) were included in this cross-sectional study. We calculated the riboflavin intake by using the food frequency questionnaire (FFQ) in combination with Chinese food composition database. Bone mineral density (BMD) was detected by an ultrasound bone densitometer. Multivariable logistic regression models were used to evaluate the relationship between dietary riboflavin intake and prevalence of osteoporosis.

**Results:**

In this population, the dietary intake of riboflavin ranged from 0.13 to 1.99 mg/d, and the proportion of abnormal BMD was 36.6%. The prevalence of osteoporosis decreased gradually with increasing quartiles of riboflavin intake, before and after adjustment for a range of confounding factors. In the final model, the multivariate-adjusted ORs (95% CI) across the quartiles of riboflavin intake were 1.00 (reference), 0.84 (0.54, 1.31), 0.59 (0.34, 1.04), and 0.47 (0.22, 0.96), respectively (*P* for trend < 0.05). In sex-disaggregated analysis, similar results to the total population were observed in women, while no significant results were found in men.

**Conclusion:**

The dietary riboflavin intake was negatively associated with the prevalence of osteoporosis. However, the association was significant in women but not in men. Our findings indicated that women are more sensitive to riboflavin intake in maintaining a normal BMD.

## 1. Introduction

Osteoporosis, a major public health problem in middle-aged and elderly people, is characterized by reduced bone mass and impaired bone microarchitecture, leading to decreased bone strength and hence increased fracture risk (1). The worldwide prevalence of osteoporosis in people aged 50 to 85 years reported as high as 21.7% (2, 3). A multi-center survey study showed that the prevalence of osteoporosis in Chinese aged > 50 years was 26.15% (4). As the proportion of the elderly population continues to increase, the health care burden caused by osteoporosis is also increasing rapidly. Therefore, it is urgent to take measures to prevent or delay the development of osteoporosis.

Bone mineral density (BMD) is an important indicator for analyzing changes in bone mass and reflecting the degree of osteoporosis. Gender, age, physical activity, smoking and drinking status, estrogen and dietary intake are all important factors that affect BMD (5, 6). Among them, dietary intake is one of controllable factors. Previous studies have confirmed the key roles of dietary calcium, phosphorus and vitamin D in maintaining BMD (7–10). Recently, some studies have demonstrated that low dietary intake of riboflavin might be a potentially modifiable risk factor for reduced BMD. An animal study found that riboflavin supplementation resulted in higher tibiae BMD and better bone strength in turkey (11). Other animal studies revealed that riboflavin deficiency increased alveolar bone loss in rats with periodontal disease and affected skeletal growth in the offspring of rats (12, 13). Moreover, animal experiments carried out in our laboratory also showed that the rats with normal riboflavin intake were 6.0% higher in whole body BMD than those with riboflavin deficiency (unpublished data). Population-based epidemiological researches have shown that riboflavin nutritional status had a significant impact on BMD in middle-aged and elderly people, especially in methylenetetrahydrofolate reductase (MTHFR) TT gene carriers, whose BMD is more sensitive to the changes in riboflavin nutritional status (14–16). However, the study conducted by Rejnmark et al. failed to find a significant effect of dietary riboflavin on BMD (17). To our knowledge, all human studies on riboflavin and bone health were conducted so far in Western countries, where riboflavin intake is generally higher (average intake ranged from 1.5 to 2.0 mg/d) (14, 16, 17). However, the mean dietary intake of riboflavin in Chinese population is around 0.80 mg/d (18), which is lower than the recommended dietary intake (1.2–1.4 mg/d) and there is a relatively high prevalence of osteoporosis in China. However, the evidence for the association between riboflavin intake and BMD is lacking in Chinese population. Thus, we conducted this cross-sectional study to investigate the relationship between dietary riboflavin intake and prevalence of osteoporosis in Chinese adults.

## 2. Materials and methods

### 2.1. Study participants

All participants in this study were recruited from the Tianjin Chronic Low-grade Systemic Inflammation and Health (TCLSIH) Cohort Study. The TCLSIH cohort study is a large dynamic cohort study focusing on the relationships between chronic low-grade systemic inflammation and the health status of a population living in Tianjin, China, for at least 5 years. The protocol of this cohort study was approved by the Institutional Review Board of Tianjin Medical University.

**FIGURE 1:**
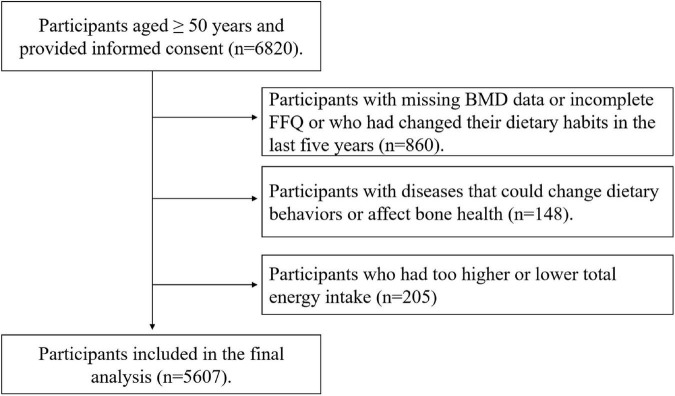
Flow diagram showing the selection of the participants.

The participants provided signed informed consent and completed questionnaire and routine health examinations. Initially, 6,820 participants aged ≥ 50 years were enrolled in this study. We excluded participants with missing BMD data or incomplete FFQ or who had changed their dietary habits in the last 5 years (*n* = 860) or participants with cancer and severe gastrointestinal diseases because they might have changed dietary behaviors. In addition, we excluded participants with hyperthyroidism or gout (*n* = 148) because these diseases could affect bone health. We also excluded participants with too higher or lower energy intake (≤400 or ≥5,000 kcal/d) (*n* = 205). Finally, 5,607 participants including 1,931 males and 3,676 females entered this study. The participant selection process was shown in Figure 1.

### 2.2. Measurement of BMD

We measured BMD by heel ultrasound. The ultrasound examination was conducted by trained investigators using an ultrasound bone densitometer (OSTEOSPACE/PEGASUS, MEDILINK, France). The subject was barefoot and the skin surface of the heel area was disinfected and then evenly coated with an appropriate amount of coupling agent. The measured foot was pressed firmly against the pedal for adjustment and fixation. The radiation beam of the scanner automatically scanned the trabecular part of the calcaneus and displayed the test results on the screen. BMD was expressed as T-score which reflects the difference from the peak BMD of normal young people of the same sex. T-score was calculated using the following formula: T-score = (BMD for the subjects–mean BMD of normal young people of the same sex)/standard deviations of the BMD of normal young people of the same sex (19, 20). The cut-off points for classifying BMD are as follows.

$$\begin{array}{cc} -1\leq T\,\text{-}\,\mathrm{score}\; & \mathrm{Normal}\,\mathrm{BMD}\,\mathrm{value}\\ -2.5<T\,\text{-}\,\mathrm{score}<-1\; & \mathrm{Low}\,\mathrm{bone}\,\mathrm{mass}\,\mathrm{or}\,\mathrm{bone}\,\mathrm{loss}\\ T\,\text{-}\,\mathrm{score}\leq -2.5\; & \mathrm{Osteoporosis} \end{array}$$

### 2.3. Calculation of riboflavin intake

Participants were asked to complete a validated semi-quantitative food frequency questionnaire (FFQ) containing 100 food items during the interview. The FFQ included seven categories ranging from “almost never eat” to “2 or more times per day” for foods and eight categories ranging from “almost never drink” to “4 or more times per day” for beverages. The total riboflavin intake was calculated by multiplying the frequency of intake of a specific portion size by the content of riboflavin in a specific food item, and then adding up the products across all food items. The content of riboflavin in food was taken from the Chinese food composition tables. An *ad hoc* computer program developed for the analysis of FFQ was used to complete this calculation process. The dietary intake of energy and other nutrients were also calculated accordingly.

The reliability and validity of the FFQ have been tested. We randomly selected 150 participants from the TCLSIH cohort and compared their questionnaire data with data from two dietary questionnaires collected 3 months apart as well as 4-day weighed dietary records (WDRs). Spearman’s correlation coefficient between two FFQs administered 3 months apart was 0.68 for energy, 0.62 to 0.79 for food items (fruits, vegetables, fish, meat, sweet foods, and beverages). Spearman’s rank correlation coefficient between the WDRs and the FFQ was 0.49 for energy, 0.35 to 0.54 for nutrients (carbohydrates, proteins, calcium, and riboflavin).

### 2.4. Assessment of other covariables

The sociodemographic variables, including age, sex, smoking status, drinking status, working status, individual, and family history of diseases, were investigated by a structured interview questionnaire. The anthropometric variables including waist circumference (WC), height and weight were measured according to a standard protocol. Body mass index (BMI) was calculated as weight in kilograms divided by height in meters squared. Blood pressure (BP) was measured at least twice by an automatic device (TM-2655, A&D Company, Ltd., Tokyo, Japan) while the participants were in a sitting position after 5 min of rest. All the participants were asked to fast overnight before collecting blood samples. Fasting blood glucose (FBG) was measured using a glucose oxidase method. High density lipoprotein cholesterol (HDL-C) was determined by the chemical precipitation method and low density lipoprotein cholesterol (LDL-C) was measured by the polyvinyl sulfuric acid precipitation method. Total cholesterol (TC) and triglyceride (TG) were measured using an automatic biochemical analyzer (Roche Cobas 8000 modular analyzer, Mannheim, Germany).

Hypertension was defined as systolic blood pressure (SBP) ≥ 140 mmHg and/or diastolic blood pressure (DBP) ≥ 90 mmHg and/or having a history of hypertension and/or using anti-hypertensive drugs (21). Diabetes was defined as FBG levels ≥ 7 mmol/L and/or having a history of diabetes and/or using anti-diabetic drugs (22). Hyperlipidemia was defined as TC ≥ 5.17 mmol/L and/or TG ≥ 1.7 mmol/L and/or LDL-C ≥ 3.37 mmol/L and/or using anti-hyperlipidemia drugs (23).

Physical activity (PA) in the most recent week was assessed by the short form of the International Physical Activity Questionnaire (IPAQ). Total PA was calculated as metabolic equivalent hours per week (MET-h/week).

The Self-Rating Depression Scale (SDS) was used to assess depressive symptoms, which comprises 20 items. Each item has four options, and each option corresponds to a different rating value. The total score ranges from 20 to 80, and higher score indicates that depressive symptoms are serious. Depression is defined as when the score is 45 (24).

### 2.5. Statistical analysis

The data were normalized by natural logarithm before statistical analysis of non-normal variables. Participant characteristics were compared using analysis of covariance (ANCOVA) for continuous variables and logistic regression analysis for categorical variables, with adjusting for age and sex. Descriptive data were presented as geometric means (95% confidence interval, CI) for continuous variables and as percentages for categorical variables.

Multivariable logistic regression models were used to assess the relationship between dietary riboflavin intake and prevalence of osteoporosis. Dietary riboflavin intake was used as independent variables, and prevalence of osteoporosis were used as dependent variables. We calculated the odds ratios (ORs) and their 95% confidence interval (CI) using the lowest riboflavin intake category as a reference. Five models were built with different levels of adjustment for potential confounding factors. Model 1 was a crude model. Model 2 was adjusted for age and sex. Model 3 was additionally adjusted for BMI. Model 4 was adjusted for variables in model 3 plus smoking status, drinking status, PA, hypertension, diabetes, hyperlipidemia, and SDS score. Model 5 was further adjusted for energy, protein, fat, carbohydrates, calcium, thiamine, vitamin B6, folate, and vitamin B12 intake in addition to all covariates mentioned above. Moreover, we performed a subgroup analysis by sex. The interaction between riboflavin intake and sex was assessed by adding cross-product terms to the multivariable logistic model.

Two sensitivity analyses were conducted to demonstrate the robustness of the study results. One sensitivity analysis was conducted using energy-adjusted riboflavin intake (nutrient residual method) instead of absolute riboflavin intake, and the other was performed after excluding participants taking calcium and multivitamin supplements.

All statistical analyses were performed using the Statistical Analysis System 9.3 edition for Windows (SAS Institute Inc., Cary, NC, USA). All tests were two-tailed and *P*-values < 0.05 were considered statistically significant.

## 3. Results

### 3.1. Characteristics of the participants

A total of 5,607 participants were included in this cross-sectional study, in which 34.4% are male. The mean age of the participants was 61.2 years, and the proportion of abnormal BMD was 36.6% (33.0% for osteopenia, 3.6% for osteoporosis). The dietary intake of riboflavin in this population ranged from 0.13 to 1.99 mg/d.

Table 1 describes the characteristics of the participants by osteoporosis status. Participants with osteoporosis were older in age on average, had higher levels of HDL-C and higher proportions of smokers and drinkers than participants without osteoporosis (*P* < 0.05 for all comparisons). However, they had lower levels of BMI and DBP, lower proportions of non-smokers and non-drinkers, and lower intakes of energy and nutrients including protein, fat, carbohydrates, calcium, thiamine, riboflavin, vitamin B6, folate, and vitamin B12 (*P* < 0.001 for all comparisons).

**TABLE 1:** Characteristics of the participants by osteoporosis status^a^ (*n* = 5,607).

Characteristics	Osteoporosis status		*P*-value^b^
	No	Yes	
No. of subjects	5,405	202	
Sex (males, %)	34.4	35.2	0.83
Age (y)	61.9 (61.7, 62.2)	67.5 (66.1, 68.9)	<0.0001
BMI (kg/m^2^)	25.0 (24.8, 25.2)	23.8 (23.2, 24.4)	0.0005
TG (mmol/L)	1.73 (1.66, 1.80)	1.44 (1.07, 1.82)	0.14
TC (mmol/L)	5.42 (5.39, 5.45)	5.40 (5.25, 5.55)	0.78
LDL-C (mmol/L)	3.29 (3.26, 3.32)	3.20 (3.05, 3.36)	0.28
HDL-C (mmol/L)	1.46 (1.45, 1.48)	1.61 (1.52, 1.69)	0.0008
SBP (mmHg)	142.8 (142.1, 143.5)	140.7 (137.3, 144.1)	0.25
DBP (mmol/L)	83.3 (82.9, 83.6)	81.3 (79.5, 83.2)	0.05
FBG (mmol/L)	5.64 (5.43, 5.84)	5.61 (4.56, 6.67)	0.97
PA (MET-h/week)^c^	59.1 (57.0, 61.1)	50.2 (39.6, 60.8)	0.11
SDS score^d^	29.0 (28.7, 29.4)	29.5 (28.2, 30.8)	0.51
**Smoking status (%)**			
Smoker	10.6	17.3	0.003
Ex-smoker	1.05	1.49	0.56
Non-smoker	88.4	81.2	0.002
**Drinker status (%)**			
Drinker	5.11	8.42	0.04
Ex-drinker	0.17	0.50	0.30
Non-drinker	94.9	91.6	0.04
Hypertension (%)	35.6	36.6	0.76
Diabetes (%)	10.8	11.4	0.79
Hyperlipidemia (%)	66.4	61.9	0.18
Energy intake (kcal/d)	1314.5 (1299.7, 1329.3)	1192.7 (1116.3, 1269.1)	0.002
**Dietary intake**			
Protein (g/d)	53.3 (52.7, 53.9)	47.1 (43.9, 50.3)	0.0002
Fat (g/d)	35.8 (35.3, 36.3)	31.2 (28.6, 33.9)	0.0008
Carbohydrate (g/d)	202.8 (200.6, 205.1)	188.0 (176.3, 199.7)	0.01
Calcium (mg/d)	411.5 (405.5, 417.5)	344.3 (313.3, 375.4)	<0.0001
Thiamine (mg/d)	0.44 (0.44, 0.45)	0.38 (0.35, 0.41)	<0.0001
Riboflavin (mg/d)	0.80 (0.79, 0.81)	0.71 (0.66, 0.76)	0.0003
Vitamin B6 (mg/d)	0.46 (0.46, 0.47)	0.39 (0.36, 0.42)	<0.0001
Folate (μg/d)	205.8 (202.7, 209.0)	163.4 (147.1, 179.6)	<0.0001
Vitamin B12 (μg/d)	0.15 (0.14, 0.16)	0.08 (0.05, 0.12)	0.0004

^a^Continuous variables are expressed as geometric means (95% confidence interval) and categorical variables are expressed as percentages. ^b^Analysis of variance or logistic regression analysis. ^c^PA, physical activity. ^d^SDS score, self-rating depression scale score.

Table 2 lists the characteristics of the participants by the quartiles of riboflavin dietary intake. With increasing riboflavin intake across quartile groups, participants had progressively higher proportion of males, lower proportion of hypertension and higher intakes of energy and nutrients (including protein, fat, carbohydrate, calcium, thiamine, riboflavin, vitamin B6, folate, and vitamin B12) (*P* < 0.0001 for all comparisons). In the second quartile, the age, HDL-C, PA, SDS score and the proportions of smokers, ex-smoker and drinkers were the highest, while TC, LDL-C, and the proportions of non-smokers and non-drinkers were the lowest (*P* < 0.001 for all comparisons). No significant difference was observed for other variables across quartile groups of riboflavin intake.

**TABLE 2:** Characteristics of the participants by riboflavin intake^a^ (*n* = 5,607).

Characteristics	Riboflavin intake				*P*-value^b^
	Q1	Q2	Q3	Q4	
No. of subjects	1,402	1,402	1,401	1,402	
Sex (males, %)	28.2	31.2	36.4	41.9	<0.0001
Age (y)	62.0 (61.4, 62.6)	62.5 (61.9, 63.1)	61.2 (60.7, 61.8)	59.0 (58.4, 59.5)	<0.0001
BMI (kg/m^2^)	25.2 (24.9, 25.4)	24.9 (24.7, 25.2)	24.7 (24.3, 25.0)	24.8 (24.2, 25.4)	0.16
TG (mmol/L)	1.77 (1.63, 1.91)	1.68 (1.54, 1.82)	1.77 (1.63, 1.91)	1.65 (1.51, 1.79)	0.54
TC (mmol/L)	5.42 (5.37, 5.48)	5.30 (5.25, 5.36)	5.45 (5.39, 5.51)	5.52 (5.46, 5.58)	<0.0001
LDL-C (mmol/L)	3.25 (3.19, 3.30)	3.15 (3.09, 3.21)	3.34 (3.28, 3.40)	3.42 (3.36, 3.48)	<0.0001
HDL-C (mmol/L)	1.48 (1.45, 1.51)	1.52 (1.49, 1.55)	1.47 (1.44, 1.50)	1.39 (1.36, 1.42)	<0.0001
SBP (mmHg)	142.4 (141.2, 143.6)	143.0 (141.7, 144.3)	143.6 (142.2, 145.0)	141.5 (139.9, 143.1)	0.26
DBP (mmol/L)	82.7 (82.0, 83.4)	83.6 (83.0, 84.3)	83.5 (82.7, 84.3)	82.8 (82.0, 83.7)	0.17
FBG (mmol/L)	5.47 (5.08, 5.87)	5.42 (5.03, 5.82)	6.03 (5.63, 6.43)	5.62 (5.22, 6.02)	0.14
PA (MET-h/week)^c^	60.4 (56.4, 64.4)	65.8 (61.8, 69.9)	57.9 (53.9, 62.0)	50.8 (46.8, 54.8)	<0.0001
SDS score^d^	28.6 (28.1, 29.1)	29.9 (29.4, 30.4)	29.2 (28.5, 29.9)	26.9 (25.8, 28.1)	<0.0001
**Smoking status (%)**					
Smoker	14.2	16.0	9.71	3.28	<0.0001
Ex-smoker	1.21	2.00	0.93	0.14	0.0007
Non-smoker	84.6	82.0	89.4	96.6	<0.0001
**Drinker status (%)**					
Drinker	4.92	7.63	5.85	2.50	0.0007
Ex-drinker	0.14	0.21	0.36	0.00	0.57
Non-drinker	95.1	92.4	94.2	97.5	0.0007
Hypertension (%)	42.2	40.7	33.5	26.1	<0.0001
Diabetes (%)	10.6	10.3	11.7	10.7	0.62
Hyperlipidemia (%)	67.9	63.3	66.9	67.1	0.85
Energy intake (kcal/d)	809.4 (791.9, 826.8)	1070.9 (1053.4, 1088.3)	1361.4 (1343.9, 1378.8)	1998.8 (1981.4, 2016.3)	<0.0001
**Nutrient intake**					
Protein (g/d)	31.1 (30.4, 31.8)	43.3 (42.5, 44.0)	55.3 (54.6, 56.0)	82.6 (81.9, 83.3)	<0.0001
Fat (g/d)	18.4 (17.8, 19.0)	28.0 (27.4, 28.7)	37.7 (37.1, 38.4)	58.4 (57.8, 59.1)	<0.0001
Carbohydrate (g/d)	134.8 (131.7, 137.8)	167.9 (164.8, 171.0)	208.5 (205.4, 211.6)	297.9 (294.8, 301.0)	<0.0001
Calcium (mg/d)	203.6 (196.9, 210.4)	303.9 (297.1, 310.7)	432.4 (425.6, 439.2)	696.3 (689.5, 703.1)	<0.0001
Thiamine (mg/d)	0.25 (0.24, 0.25)	0.35 (0.34, 0.36)	0.46 (0.45, 0.47)	0.71 (0.70, 0.72)	<0.0001
Riboflavin (mg/d)	0.42 (0.41, 0.43)	0.65 (0.64, 0.65)	0.85 (0.84, 0.85)	1.28 (1.27, 1.29)	<0.0001
Vitamin B6 (mg/d)	0.27 (0.26, 0.28)	0.37 (0.36, 0.37)	0.48 (0.47, 0.49)	0.73 (0.72, 0.74)	<0.0001
Folate (μg/d)	110.0 (105.8, 114.2)	154.2 (150.0, 158.3)	211.2 (207.0, 215.3)	341.9 (337.8, 346.1)	<0.0001
Vitamin B12 (μg/d)	0.07 (0.05, 0.08)	0.10 (0.08, 0.11)	0.15 (0.14, 0.17)	0.27 (0.25, 0.28)	<0.0001

^a^Continuous variables are expressed as geometric means (95% confidence interval) and categorical variables are expressed as percentages. ^b^Analysis of variance or logistic regression analysis. ^c^PA, physical activity. ^d^SDS score, self-rating depression scale score.

### 3.2. Relationship between riboflavin intake and prevalence of osteoporosis

**FIGURE 2:**
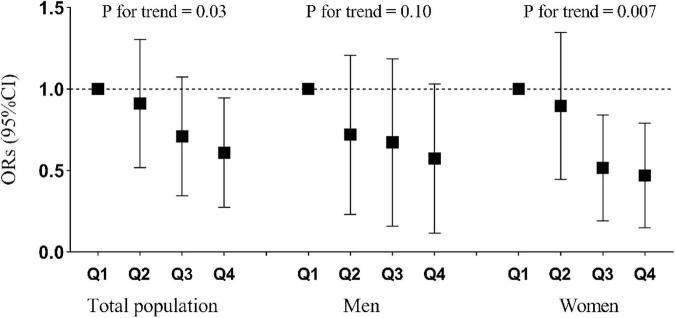
Relationship between quartiles of riboflavin intake and prevalence of osteoporosis stratified by total population, men, and women. Adjusted for age, sex, BMI, smoking status, drinking status, physical activity, hypertension, diabetes, hyperlipidemia, self-rating depression scale score, energy intake, and calcium intake.

As shown in Table 3, the number of participants with osteoporosis decreased gradually with increasing quartiles of riboflavin intake. In model 1, no confounding factor was adjusted, and the ORs (95% CI) of osteoporosis across the increasing quartiles of riboflavin intake were 1.00 (reference), 0.93 (0.65, 1.34), 0.71 (0.48, 1.04), and 0.54 (0.35, 0.82), respectively (*P* for trend = 0.002). After progressive adjustment for the confounding factors of sex, age, BMI, smoking status, drinking status, physical activity, hypertension, diabetes, hyperlipidemia, SDS score, energy, protein, fat, carbohydrates, calcium, thiamine, vitamin B6, folate, and vitamin B12 intake, the results did not change substantially. In model 5, the multivariate-adjusted ORs (95% CI) were 1.00 (reference), 0.84 (0.54, 1.31), 0.59 (0.34, 1.04), and 0.47 (0.22, 0.96), respectively (*P* for trend = 0.03). There was an inverse association between riboflavin intake and osteoporosis prevalence. Moreover, the OR for the prevalence of osteoporosis in the highest quartile of riboflavin intake was significantly lower than that in the lowest quartile of riboflavin intake (Figure 2).

**TABLE 3:** Relationship between quartiles of riboflavin intake and prevalence of osteoporosis in total population.

	Riboflavin intake				*P* for trend^a^
	Q1	Q2	Q3	Q4	
**All (*n* = 5,607)**					
Riboflavin intake range (mg/d)	0.13–0.55	0.55–0.74	0.74–0.98	0.98–1.99	
Cases/subjects	63/1,402	59/1,402	45/1,401	35/1,402	
Model 1^b^	1.00 (reference)	0.93 (0.65, 1.34)^c^	0.71 (0.48, 1.04)	0.54 (0.35, 0.82)	0.002
Model 2^d^	1.00 (reference)	0.90 (0.63, 1.30)	0.72 (0.48, 1.06)	0.59 (0.38, 0.90)	0.008
Model 3^e^	1.00 (reference)	0.89 (0.62, 1.29)	0.73 (0.48, 1.08)	0.63 (0.40, 0.98)	0.03
Model 4^f^	1.00 (reference)	0.88 (0.60, 1.27)	0.72 (0.48, 1.07)	0.62 (0.40, 0.97)	0.02
Model 5^g^	1.00 (reference)	0.84 (0.54, 1.31)	0.59 (0.34, 1.04)	0.47 (0.22, 0.96)	0.03

^a^Multiple-logistic regression analysis. ^b^Model 1 was crude model. ^c^Odds ratio (95% confidence interval) (all such values). ^d^Model 2 was adjusted for age and sex. ^e^Model 3 was adjusted for variables in model 2 plus body mass index. ^f^Model 4 was adjusted for variables in model 3 plus smoking status, drinking status, physical activity, hypertension, diabetes, hyperlipidemia, and self-rating depression scale score. ^g^Model 5 was adjusted for variables in model 4 plus energy, protein, fat, carbohydrates, calcium, thiamine, vitamin B6, folate, and vitamin B12 intake.

The interaction between riboflavin intake and sex was not statistically significant (*P* for interaction = 0.35). The results of the sex-disaggregated analysis (Table 4) revealed that the associations between riboflavin intake and prevalence of osteoporosis differed between men and women. In men, no significant results were found in any of the models. In women, before and after adjustment for variables including sex, age, BMI, smoking status, drinking status, physical activity, hypertension, diabetes, hyperlipidemia, SDS score, energy, protein, fat, carbohydrates, calcium, thiamine, vitamin B6, folate, and vitamin B12 intake, the riboflavin intake was consistently, significantly and negatively associated with the prevalence of osteoporosis (*p* < 0.05). Moreover, the OR for the prevalence of osteoporosis in the highest and third quartiles of riboflavin intake was significantly lower than that in the lowest quartile of riboflavin intake (Figure 2).

**TABLE 4:** Relationship between quartiles of riboflavin intake and prevalence of osteoporosis stratified by sex.

	Riboflavin intake				*P* for trend^a^
	Q1	Q2	Q3	Q4	
**Men (*n* = 1,931)**					
Riboflavin intake range (mg/d)	0.16–0.59	0.59–0.79	0.79–1.05	1.05–1.99	
Cases/subjects	19/483	20/483	18/482	14/483	
Model 1^b^	1.00 (reference)	1.06 (0.55, 2.02)^c^	0.95 (0.49, 1.84)	0.73 (0.36, 1.46)	0.34
Model 2^d^	1.00 (reference)	1.03 (0.54, 1.97)	0.95 (0.49, 1.84)	0.76 (0.37, 1.53)	0.42
Model 3^e^	1.00 (reference)	1.02 (0.53, 1.95)	0.95 (0.49, 1.85)	0.79 (0.37, 1.63)	0.51
Model 4^f^	1.00 (reference)	0.97 (0.50, 1.88)	0.93 (0.47, 1.83)	0.77 (0.36, 1.60)	0.49
Model 5^g^	1.00 (reference)	0.59 (0.27, 1.29)	0.45 (0.18, 1.14)	0.42 (0.14, 1.28)	0.10
**Women (*n* = 3,676)**					
Riboflavin intake range (mg/d)	0.13–0.53	0.53–0.71	0.71–0.94	0.94–1.99	
Cases/subjects	47/919	42/919	23/919	19/919	
Model 1^b^	1.00 (reference)	0.89 (0.58, 1.36)	0.48 (0.28, 0.78)	0.39 (0.22, 0.66)	<0.0001
Model 2^d^	1.00 (reference)	0.87 (0.56, 1.34)	0.50 (0.29, 0.82)	0.45 (0.25, 0.76)	0.0006
Model 3^e^	1.00 (reference)	0.86 (0.55, 1.32)	0.50 (0.29, 0.84)	0.47 (0.26, 0.83)	0.003
Model 4^f^	1.00 (reference)	0.82 (0.53, 1.27)	0.50 (0.29, 0.83)	0.46 (0.25, 0.81)	0.002
Model 5^g^	1.00 (reference)	0.81 (0.48, 1.36)	0.42 (0.21, 0.83)	0.30 (0.11, 0.79)	0.007

^a^Multiple-logistic regression analysis. ^b^Model 1 was crude model. ^c^Odds ratio (95% confidence interval) (all such values). ^d^Model 2 was adjusted for age. ^e^Model 3 was adjusted for variables in model 2 plus body mass index. ^f^Model 4 was adjusted for variables in model 3 plus smoking status, drinking status, physical activity, hypertension, diabetes, hyperlipidemia, and self-rating depression scale score. ^g^Model 5 was adjusted for variables in model 4 plus energy, protein, fat, carbohydrates, calcium, thiamine, vitamin B6, folate, and vitamin B12 intake.

### 3.3. Sensitivity analysis

In sensitivity analysis with energy-adjusted riboflavin intake as the exposure, we observed similar results (Supplementary Table 1). When participants taking calcium and multivitamin supplements were excluded from the analyses, the multivariate-adjusted ORs (95% CIs) across the quartiles of riboflavin intake were 1.00 (reference), 0.82 (0.52, 1.29), 0.62 (0.35, 1.10), and 0.47 (0.22, 0.98), *P* for trend = 0.04 (Supplementary Table 2). These data indicate that the results do not change substantially after excluding participants taking calcium and multivitamin supplements.

## 4. Discussion

In this cross-sectional study, we found that dietary riboflavin intake was negatively associated with the prevalence of osteoporosis in Chinese adults. Moreover, the prevalence of osteoporosis was significantly lower in the highest quartile of riboflavin intake than in the lowest quartile of riboflavin intake. However, the association between riboflavin intake and the prevalence of osteoporosis showed different outcomes in men and women. No significant trends were found in men. In women, riboflavin intake was negatively associated with the prevalence of osteoporosis, and the prevalence of osteoporosis in the highest and third quartiles of riboflavin intake was significantly lower than in the lowest quartile of riboflavin intake. To our knowledge, this is the first study on the relationship between riboflavin dietary intake and prevalence of osteoporosis in a Chinese population.

Our findings suggested that low riboflavin intake was detrimental to bone health, which is to some extent consistent with the results reported by several previous studies. In an elderly population from the Rotterdam study, it was found that a significant and independent positive association between dietary riboflavin intake and BMD (15). Differently from our results, this study did not analyze the association by men and women separately. In our multivariate adjustment process for the analysis of the association between riboflavin intake and prevalence of osteoporosis, we found that men and women showed different trends, leading us to speculate that women are more sensitive to riboflavin intake in terms of the prevalence of osteoporosis. Another longitudinal study, also from the Rotterdam population, observed a significant effect of riboflavin intake on fracture risk only in women, confirming gender differences in the association between dietary riboflavin intake and BMD (25). Middle-aged and older women in the perimenopausal period, when estrogen levels decline substantially and bone damage increases, are more sensitive to the factors that affect bone health (26–28). In our study, the limited sample size of men may result in low statistical power among men, which may also account for the gender differences. To date, only one study conducted in women failed to find a significant effect of riboflavin intake on BMD (17). However, the average intake of riboflavin in this population was 2.7 mg/d, which was much higher than the recommended intake levels.

The study by Yazdanpanah et al. confirmed the important influence of MTHFR gene polymorphisms on the association between dietary riboflavin intake and BMD (25). In addition, two women-only studies found that low riboflavin intake resulted in significantly lower BMD in individuals who were MTHFR TT genotype carriers (14, 16). Other two studies have shown that MTHFR TT genotype carriers were more sensitive to the changes in riboflavin nutritional status (29, 30). However, the frequency of the MTHFR TT genotype was 5.5% in the Asian population, much lower than 11.9% in the American population and 11.6% in the European population (31). Moreover, one study showed that MTHFR TT genotype was not associated with BMD in Chinese population (32). Therefore, this genotype may not be an important factor influencing the relationship between riboflavin intake and prevalence of osteoporosis in Chinese population.

Although the exact mechanisms of the beneficial effects of riboflavin on BMD have not been clarified, there are several hypotheses proposed as follows. First, riboflavin may impact on BMD through its effect on homocysteine levels. Flavin adenine dinucleotide (FAD), one of the biologically active forms of riboflavin, is involved in the metabolism of homocysteine as a cofactor (1, 33). Two studies demonstrated that homocysteine concentration was negatively correlated with BMD (34, 35). Araki et al. reported that riboflavin supplementation decreased homocysteine levels in the body (36). Second, as a coenzyme of several key enzymes required in multiple processes such as energy metabolism and oxidative stress (37–39), riboflavin may have direct impacts on the functions of osteoblasts and osteoclasts, which in turn affect BMD.

The present study has several strengths. First, the validated FFQ we used provides the most comprehensive range of foods consumed daily by Chinese people, which helps to reflect nutritional intake more accurately. Second, well-trained staff and rigorous inclusion and exclusion criteria for participants make our data more reliable. Finally, this is the first study reporting the relationship between dietary riboflavin intake and prevalence of osteoporosis in a Chinese adult population, which differs nutritionally and genetically from western populations. However, this study also has some limitations. First, we calculated the dietary intake of riboflavin, but the bioavailability of riboflavin was not assessed. Future studies are needed to validate the bioavailability of dietary riboflavin by measuring specific biomarkers. Second, we measured BMD using ultrasound rather than dual energy X-rays absorptiometry (DXA). Ultrasound measurements had a lower sensitivity but a higher specificity for diagnosing osteoporosis compared to DXA measurements (40). There is a good consistency in the results between the two diagnostic tools (41, 42). Moreover, ultrasound bone densitometry is safer without radiation, portable and suitable in screening for osteoporosis in a large sample of people (43). Third, dietary intake was measured by using a self-report questionnaire, therefore recall bias might exist. However, our FFQ has good reliability and validity, which makes the data reliable. Fourth, the present study was a cross-sectional study, and the causality between riboflavin intake and osteoporosis could not be proven. Finally, we did not test participants for different genotypes though the frequency of MTHFR TT genotype was lower in Chinese population. More in-depth studies on the polymorphisms in BMD-related genes are needed to determine the significance of their impacts on BMD.

## 5. Conclusion

The dietary riboflavin intake was negatively associated with the prevalence of osteoporosis in Chinese adult population. In addition, the relationship was significant in women but not in men. Our findings indicated that women are more sensitive to riboflavin intake in maintaining a normal BMD. Further *in vitro* or animal experiments are needed to explore the sex-specific mechanisms of the actions of riboflavin on BMD in detail.

## Data availability statement

The raw data supporting the conclusions of this article will be made available by the authors, without undue reservation.

## Ethics statement

The studies involving human participants were reviewed and approved by the Institutional Review Board of Tianjin Medical University. The patients/participants provided their written informed consent to participate in this study.

## Author contributions

MW, KN, and CG designed the research. MW, HW, XW, and YG managed and analyzed the data. MW, GM, QZ, LL, JZ, SS, QJ, KS, and WG conducted the research. MW, KN, and CG wrote the manuscript. ZY, KN, and CG had primary responsibility for final content. All authors approved the final version of the manuscript.
