# Supplementary material for: There is a significantly inverse relationship between dietary riboflavin intake and prevalence of osteoporosis in women but not in men: Results from the TCLSIH cohort study
Source: Front Nutr. 2023 Feb 7;10:1112028. doi: 10.3389/fnut.2023.1112028 (PMC9941537; doi:10.3389/fnut.2023.1112028)
Supplement: Supplementary file 1 [file Table_1.docx]

**Table S1** Relationship between quartiles of energy-adjusted riboflavin intake and prevalence of osteoporosis.

|  | Adjusted riboflavin intake | | | | P for trend |
| --- | --- | --- | --- | --- | --- |
|  | Q1 | Q2 | Q3 | Q4 |  |
| All (n=5607) |  |  |  |  |  |
| Cases/subjects | 50/1402 | 59/1402 | 63/1401 | 30/1402 |  |
| Multivariate-adjusted model^a^ | 1.00 (reference) | 1.13 (0.76, 1.69) | 1.19 (0.80, 1.77) | 0.61 (0.37, 0.98) | 0.11 |
| Men (n=1931) |  |  |  |  |  |
| Cases/subjects | 19/483 | 17/483 | 22/482 | 13/483 |  |
| Multivariate-adjusted model | 1.00 (reference) | 0.81 (0.4, 1.61) | 1.03 (0.54, 2.00) | 0.61 (0.28, 1.27) | 0.33 |
| Women (n=3676) |  |  |  |  |  |
| Cases/subjects | 34/919 | 40/919 | 39/919 | 18/919 |  |
| Multivariate-adjusted model | 1.00 (reference) | 1.14 (0.71, 1.85) | 1.03 (0.62, 1.71) | 0.54 (0.29, 0.99) | 0.07 |

^a^ Multivariate-adjusted model was adjusted for age, sex, body mass index, smoking status, drinking status, physical activity, hypertension, diabetes, hyperlipidemia and self-rating depression scale score, energy, protein, fat, carbohydrates, calcium, thiamine, vitamin B6, folate and vitamin B12 intake.

**Table S2** Relationship between quartiles of riboflavin intake and prevalence of osteoporosis in sensitivity analysis.

|  | Riboflavin intake | | | | P for trend^a^ |
| --- | --- | --- | --- | --- | --- |
|  | Q1 | Q2 | Q3 | Q4 |  |
| All (n=5273) |  |  |  |  |  |
| Riboflavin intake range (mg/d) | 0.13-0.55 | 0.55-0.73 | 0.73-0.97 | 0.97-1.99 |  |
| Cases/subjects | 59/1319 | 55/1318 | 42/1317 | 33/1319 |  |
| Multivariate-adjusted model | 1.00 (reference) | 0.82 (0.52, 1.29) | 0.62 (0.35, 1.10) | 0.47 (0.22, 0.98) | 0.04 |
| Men (n=1858) |  |  |  |  |  |
| Riboflavin intake range (mg/d) | 0.16-0.59 | 0.59-0.78 | 0.78-1.03 | 1.04-1.99 |  |
| Cases/subjects | 18/465 | 20/464 | 17/464 | 12/465 |  |
| Multivariate-adjusted model | 1.00 (reference) | 0.62 (0.28, 1.39) | 0.43 (0.17, 1.12) | 0.41 (0.13, 1.26) | 0.12 |
| Women (n=3415) |  |  |  |  |  |
| Riboflavin intake range (mg/d) | 0.13-0.53 | 0.53-0.70 | 0.70-0.93 | 0.93-1.99 |  |
| Cases/subjects | 44/854 | 41/854 | 23/853 | 19/854 |  |
| Multivariate-adjusted model | 1.00 (reference) | 0.86 (0.51, 1.47) | 0.46 (0.22, 0.94) | 0.29 (0.11, 0.78) | 0.03 |

^a^ Multivariate-adjusted model was adjusted for age, sex, body mass index, smoking status, drinking status, physical activity, hypertension, diabetes, hyperlipidemia and self-rating depression scale score, energy, protein, fat, carbohydrates, calcium, thiamine, vitamin B6, folate and vitamin B12 intake.
